# Supplementary material for: Activation of P2X7 Receptor Mediates the Abnormal Ovulation Induced by Chronic Restraint Stress and Chronic Cold Stress
Source: Biology (Basel). 2024 Aug 15;13(8):620. doi: 10.3390/biology13080620 (PMC11351884; doi:10.3390/biology13080620)
Supplement: Supplementary file 1 [file biology-13-00620-s001.zip › biology-3104073-supplementary.pdf]

For Figure 3B P2X7

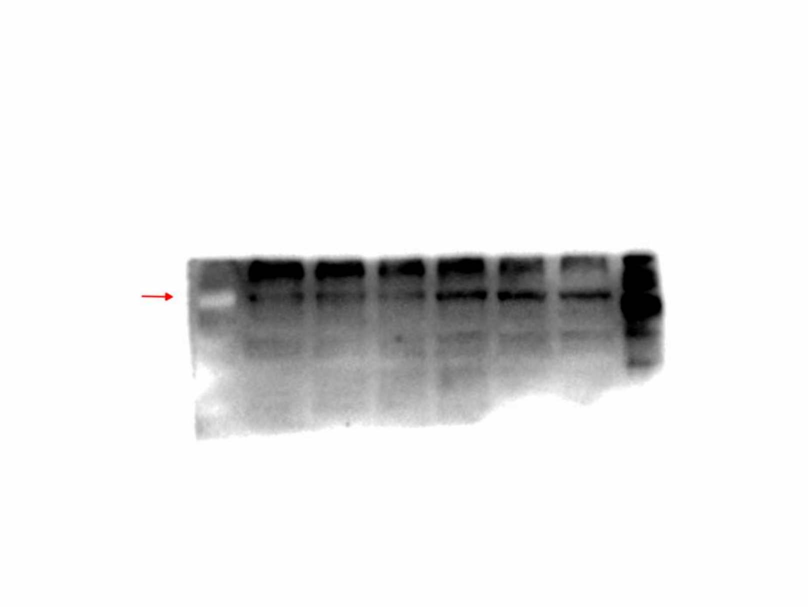

For Figure 3B β-actin

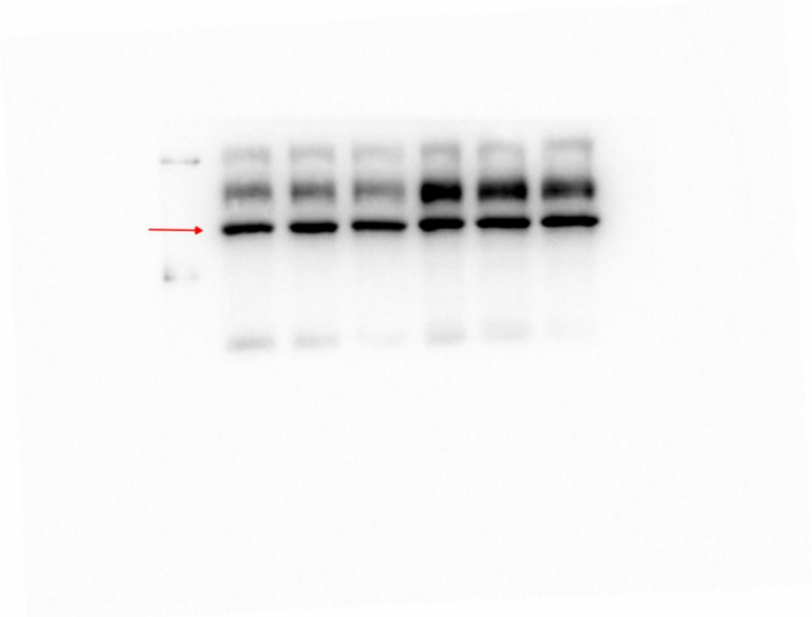

Supplementary Figure S1 Full unedited Western blot images for Figure 3B P2X7 and β-actin.

For Figure 3C P2X7

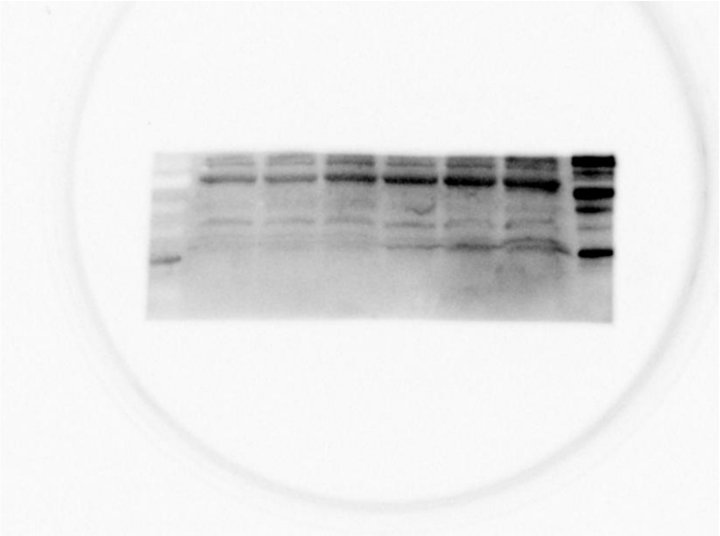

For Figure 3C β-actin

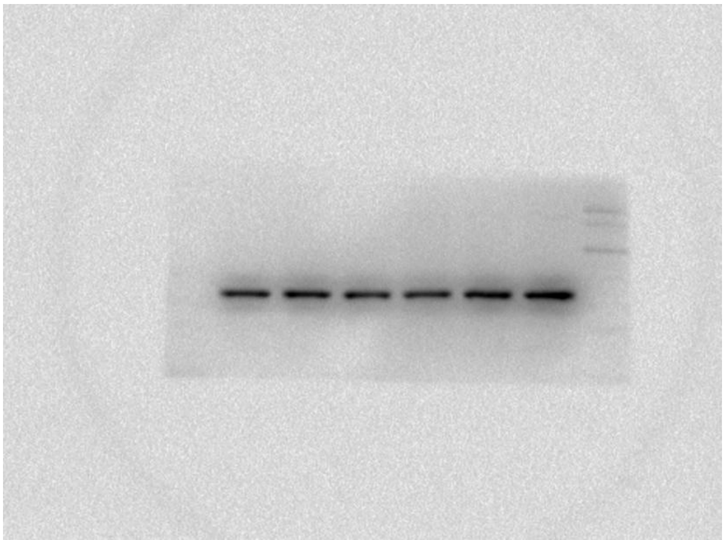

Supplementary Figure S2 Full unedited Western blot images for Figure 3C P2X7 and β-actin.

For Figure 5B MMP2

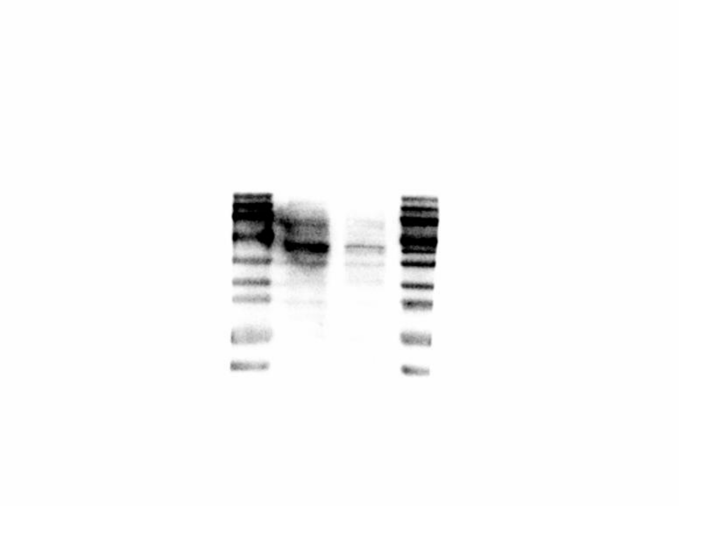

For Figure 5B TGF β 1

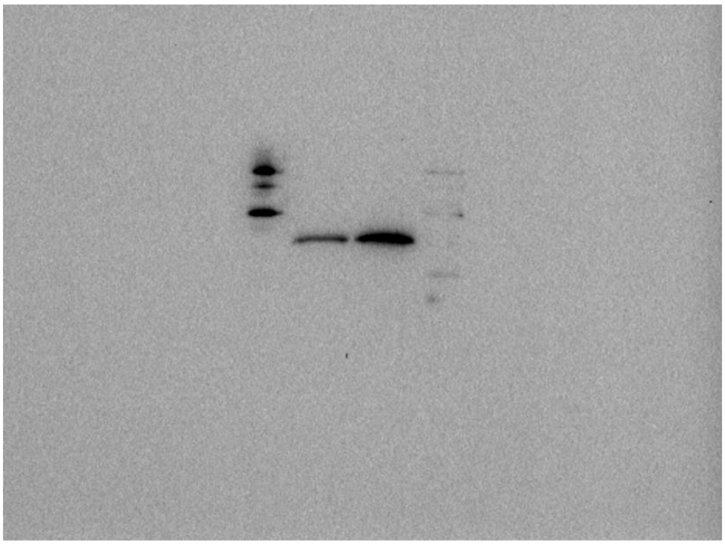

For Figure 5B β-actin

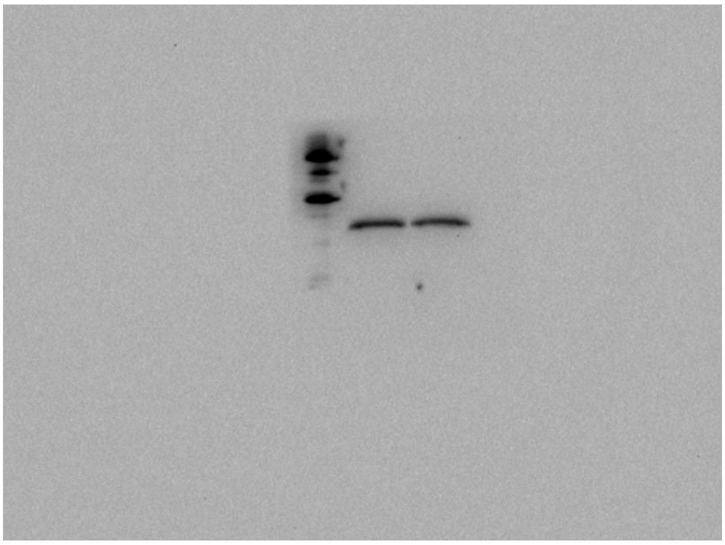

Supplementary Figure S3 Full unedited Western blot images for Figure 5B MMP2、 TGFβ1 and β-actin.

For Figure 5C MMP2

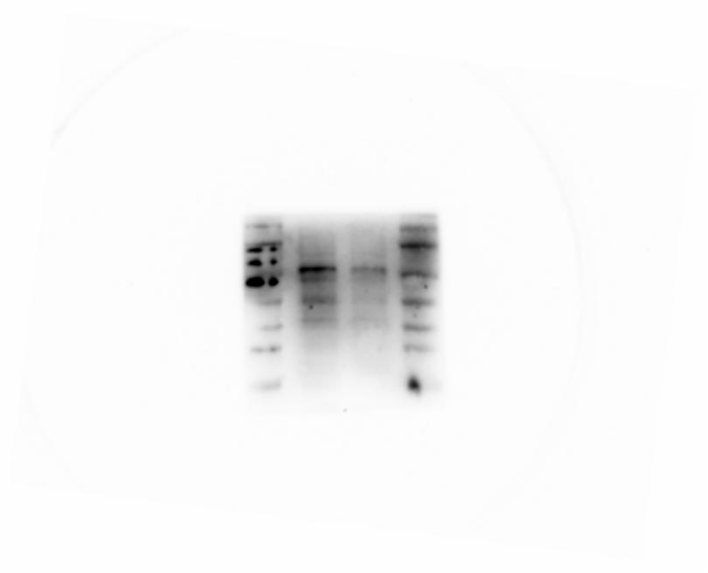

For Figure 5C TGF β 1

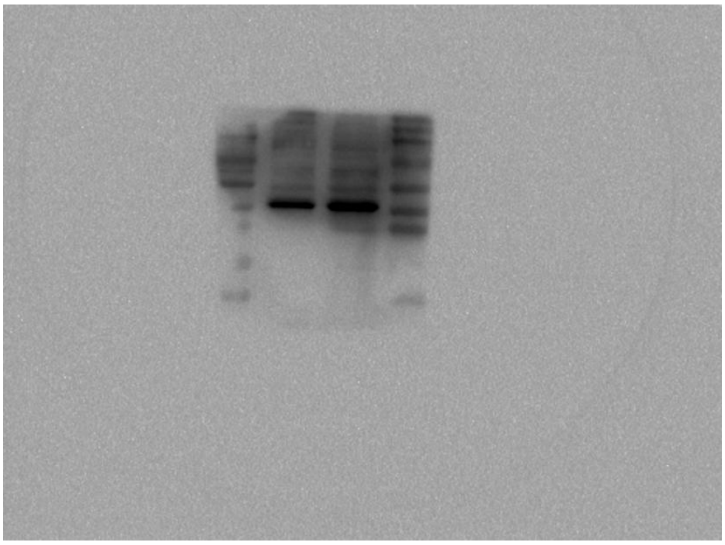

For Figure 5C β-actin

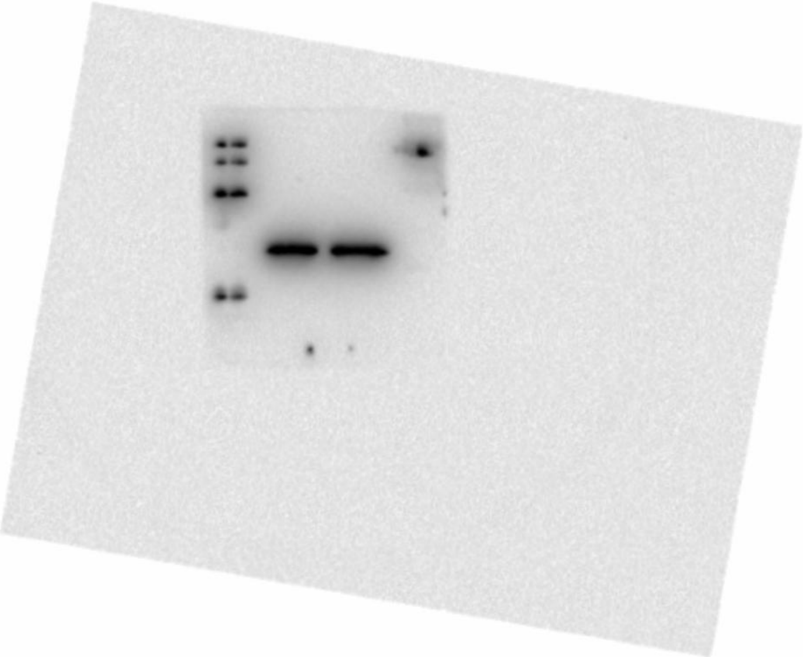

Supplementary Figure S4 Full unedited Western blot images for Figure 5C MMP2、 TGF β 1 and β-actin.

For Figure 5D MMP2

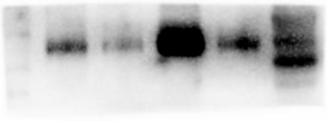

con

ZFor Figure 5D TGF β1

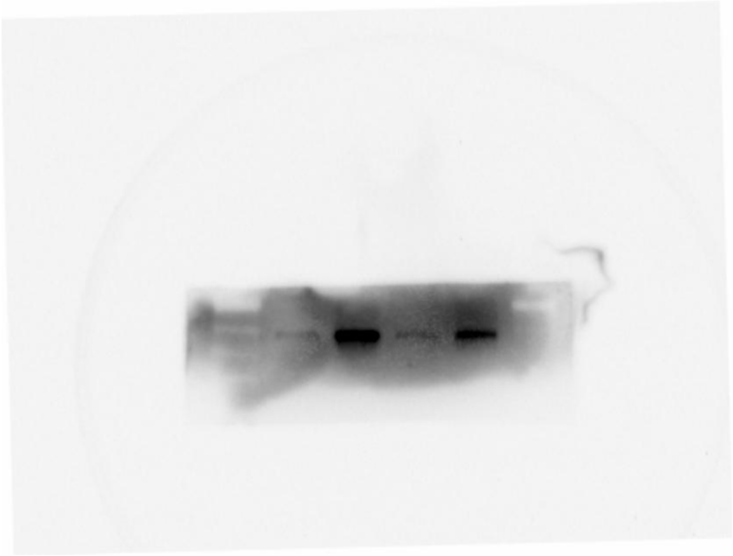

For Figure 5D β-actin

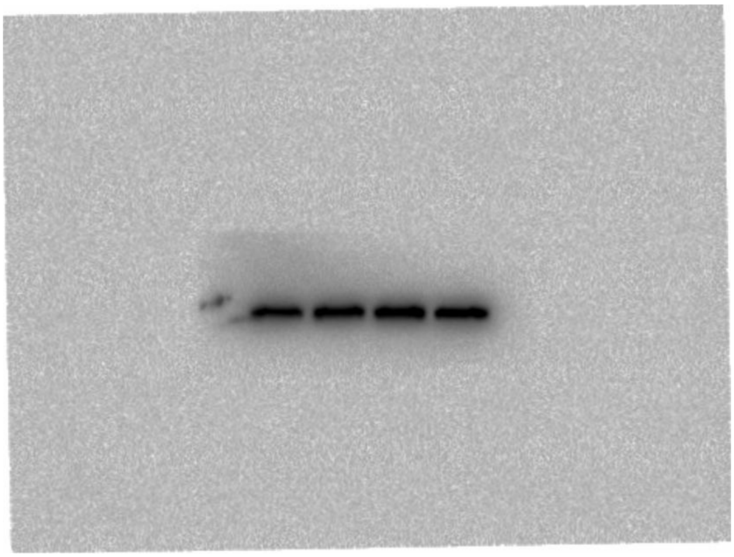

Supplementary Figure S5 Full unedited Western blot images for Figure 5D MMP2、 TGFβ1 and β-actin.
